# Metabolic characteristics of dominant microbes and key rare species from an acidic hot spring in Taiwan revealed by metagenomics

**DOI:** 10.1186/s12864-015-2230-9

**Published:** 2015-12-03

**Authors:** Kuei-Han Lin, Ben-Yang Liao, Hao-Wei Chang, Shiao-Wei Huang, Ting-Yan Chang, Cheng-Yu Yang, Yu-Bin Wang, Yu-Teh Kirk Lin, Yu-Wei Wu, Sen-Lin Tang, Hon-Tsen Yu

**Affiliations:** Department of Life Science, National Taiwan University, Taipei, 10617, Taiwan Republic of China; Division of Biostatistics & Bioinformatics, Institute of Population Health Sciences, National Health Research Institutes, Zhunan Town, Miaoli County 35053, Taiwan Republic of China; Molecular Microbiology and Microbial Pathogenesis Program, Division of Biology and Biomedical Science, Washington University in St. Louis, St. Louis, MO 63130 USA; Biodiversity Research Center, Academia Sinica, Taipei, 11529, Taiwan Republic of China; Institute of Information Science, Academia Sinica, Taipei 11529, Taiwan Republic of China; Institute of Ecology and Evolutionary Biology, National Taiwan University, Taipei, 10617, Taiwan Republic of China; Joint BioEnergy Institute, Emeryville, CA 94608 USA; Physical Biosciences Division, Lawrence Berkeley National Laboratory, Berkeley, CA 94720 USA; Degree Program of Genome and Systems Biology, National Taiwan University and Academia Sinica, Taipei, 10617, Taiwan Republic of China

## Abstract

**Background:**

Microbial diversity and community structures in acidic hot springs have been characterized by 16S rRNA gene-based diversity surveys. However, our understanding regarding the interactions among microbes, or between microbes and environmental factors, remains limited.

**Results:**

In the present study, a metagenomic approach, followed by bioinformatics analyses, were used to predict interactions within the microbial ecosystem in Shi-Huang-Ping (SHP), an acidic hot spring in northern Taiwan. Characterizing environmental parameters and potential metabolic pathways highlighted the importance of carbon assimilatory pathways. Four distinct carbon assimilatory pathways were identified in five dominant genera of bacteria. Of those dominant carbon fixers, *Hydrogenobaculum* bacteria outcompeted other carbon assimilators and dominated the SHP, presumably due to their ability to metabolize hydrogen and to withstand an anaerobic environment with fluctuating temperatures. Furthermore, most dominant microbes were capable of metabolizing inorganic sulfur-related compounds (abundant in SHP). However, *Acidithiobacillus ferrooxidans* was the only species among key rare microbes with the capability to fix nitrogen, suggesting a key role in nitrogen cycling. In addition to potential metabolic interactions, based on the 16S rRNAs gene sequence of *Nanoarchaeum*-related and its potential host *Ignicoccus*-related archaea, as well as sequences of viruses and CRISPR arrays, we inferred that there were complex microbe-microbe interactions.

**Conclusions:**

Our study provided evidence that there were numerous microbe-microbe and microbe-environment interactions within the microbial community in an acidic hot spring. We proposed that *Hydrogenobaculum* bacteria were the dominant microbial genus, as they were able to metabolize hydrogen, assimilate carbon and live in an anaerobic environment with fluctuating temperatures.

**Electronic supplementary material:**

The online version of this article (doi:10.1186/s12864-015-2230-9) contains supplementary material, which is available to authorized users.

## Background

Microbial diversity surveys based on 16S rRNA gene are the most common culture-independent method to characterize composition of a microbial community and to compare microbial diversity among habitats [1]. This method has been widely used to characterize microbial community structure in a variety of acidic hot springs in diverse locations, including the Azores Islands in the North Atlantic Ocean [2], Colombian Andes [3], Iceland [4], Lassen Volcanic National Park [5, 6], Montserrat in the Caribbean sea [7], New Mexico [8], Philippines [9], St. Luica in the Lesser Antilles [10], Tengchong [11, 12], Tibet [13], West Java-Indonesia [14], and Yellowstone National Park [15, 16]. Overall, there was evidence that microbial communities in hot springs were closely linked to local environmental conditions. For example, *Sulfolobus* and *Metallosphaera,* two microbial taxa that metabolize sulfur-containing compounds, were dominant organisms in sulfur-rich hot springs [14].

Although characterizing composition of microbial communities could provide insights regarding potential metabolic interactions among microbes or between microbes and environmental factors, additional methods are required for more comprehensive understanding. For example, a metagenomic approach could be used to characterize potential metabolic activities of microbial communities. In that regard, Jiménez and coworkers successfully identified several key genes involved in metabolism of nitrogen (e.g., *narGHI*, *nirS*, *norBCDQ* and *nosZ*) and sulfur (e.g., *cysDN*, *cysNC* and *aprA*) in a microbial community in El Coquito spring, National Nature Park Los Nevados, Colombian Andes, Columbia [17]. Furthermore, in a comparative metagenomic study, Inskeep et al. characterized diverse metabolic strategies related to geochemical characteristics of two acidic hot springs (Crater Hills and Norris Geysers Basin) in Yellowstone National Park [18]. In contrast to the El Coquito spring, microbes in Crater Hills and Norris Geysers Basin adopted non-photosynthetic carbon assimilation pathways (reductive citrate cycle), apparently because water temperatures of these springs (>65 °C) approached upper temperature limits for photosynthesis [19].

Taiwan, located on the “Ring of Fire” in the West Pacific (with copious geothermal activity), is well suited for studying interactions between thermophiles and environmental factors. Tatun Volcanic Group (TVG) area (ca. 400 km^2^) in northern Taiwan comprises the largest volcanic group (>20 volcanoes) on the island. Volcanic activity started 2.8–2.5 Ma ago, with the last massive explosive event between 0.8 and 0.2 Ma ago [20]. The TVG has a ubiquitous smell of sulfuric gases, solfataras rimmed with sulfur crystals, and some sulfur mines [21]. Hot springs in this area are primarily of meteoric origin, surfacing after being heated by geothermal energy [22, 23]. Typical hot springs in the TVG area, such as Shi-Huang-Ping (SHP; also denoted Szehuangtzeping in some reports), are highly acidic (pH ~ 2.5) due to dissolved inorganic sulfur-containing compounds, and water temperature ranges from ~ 50 to 85 °C [21].

Well-documented geological features make SHP ideal for conducting a metagenomic study to characterize metabolic potential of the microbial community in acidic hyperthermal environments and relationships between the microbial community and local geochemistry. In reports that used 16S rRNA gene-based diversity surveys, *Hydrogenobaculum* was the dominant microbe at two acidic hot springs in the TVG area [24]. Furthermore, *Hydrogenobaculum*-dominant features were reported in two acidic hot springs in Yellowstone National Park (Dragon Spring and One Hundred Spring; [25, 26]). However, microbe-microbe and microbe-environment interactions within the SHP ecosystem have not been characterized.

In this study, a metagenomic approach was used to elucidate putative interactions within an acidic hot spring ecosystem. Metabolic interactions were predicted by searching metagenomic data against the KEGG database and information derived from the literature. Analyzing microbial community structure revealed potential interactions between *Ignicoccus* and an archaeal parasite *Nanoarchaea.* Furthermore, based on CRISPR array analysis, there were also potential microbe-virus interactions.

## Results and discussion

### Hydrological parameters of SHP

Limnological parameters of the SHP are shown (Table 1). Temperature and pH of the sample were 69 °C and 2.5, respectively. Concentrations of several ions (Cl^−^, HCO^3−^, Ca^2+^, Mg^2+^, K^+^ and Na^+^) were low, as was that of dissolved organic carbon (~1 mg/L). In addition, concentrations of sulfate (378 mg/L), hydrogen sulfide (52.7 mg/L) thiosulfate (0.12 mg/L) and elemental sulfur (0.50 mg/L) in SHP water were also determined.

**Table 1:** Geochemical and physical parameters of SHP hot spring water

Parameter^a^	Current study	Song et al., 2005^b^ [21]
pH	2.5	2.77 – 3.25
Temperature (°C)	69.0	49.8 – 85.1
TDS	707	177 – 1674
EC (μS/cm^2^)	1760	266 – 1039
ORP (mv)	−62	–
DOC	1.0	–
HCO_3_^2−^	<0.03	L
NO^3−^	–	L – 0.9
PO_4_^3−^	–	L
Cl^−^ and other halides	9.3	L (F^−^) / 4.93 – 10.3 (Cl^−^) / L – 3.78 (Br^−^)
SO_4_^2−^	378	100 – 450
S^0^	0.50	–
H_2_S	52.7	–
S_2_O_3_^2−^	0.12	–
Fe^2+^ and Fe^3+^	111	1.8 – 54.9 (Fe^2+^)
Ca^2+^	1.32	5.8 – 977
Na^+^	12.0	L – 13.4
Mg^2+^	1.21	1.21 – 9.70
K^+^	2.17	1.40 – 6.00
Al^3+^	19.0	0.70 – 17.3
Total As	0.0012	–

“L” represented concentrations below detection limits

*TDS* total dissolved solids, *EC* electrical conductivity, *ORP* oxidation/reduction potential, *DOC* dissolved organic carbon

^a^Units were mg/L for all end points except pH

^b^Source: Geological survey and potential application of hot springs and geothermal energy of Yangmingshan (in Chinese); monthly report of environmental parameters in the SHP acidic hot springs in 2005

### The abundant genera in SHP

**Fig. 1:**
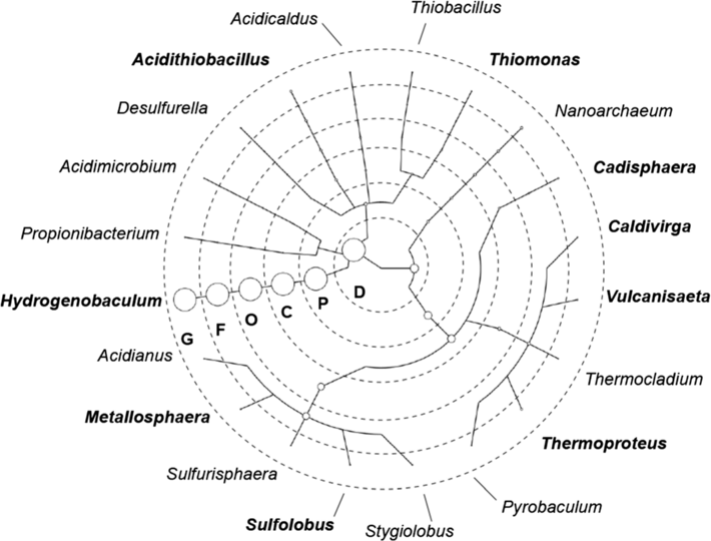
Phylogenetic tree generated from top 20 most abundant genera identified with 16S rRNA gene-based diversity survey. Diameters of circles are proportional to abundances; the smallest circles represent *RA*_*16S*_ ~ 0.02 %, whereas the largest circle (*Hydrogenobaculum*) represent *RA*_*16S*_ = 86.3 %. Letters within each circle represent taxonomy levels: domain (D), phylum (P), class (C), order (O), family (F) and genus (G). Genera names are outside the circle. Genera names in bold were common genera (top 20 most abundant and information-rich genera)

The top 20 abundant genera (eight bacterial and 12 archaeal genera; Fig. 1) were selected (from 16S rRNA gene-based diversity surveys) for phylogenetic analyses. Although there were more archaeal than bacterial genera in the top 20, bacteria clearly dominated the microbial community in SHP, based on relative abundance of 16S rRNA. In that regard, *Hydrogenobaculum* bacteria accounted for 86.30 % of *RA*_*16S*_ (relative abundance in 16S rRNA gene-based diversity survey), whereas the second most abundant genus, *Nanoarchaeum* (an archaeal genus) only accounted for approximately 0.99 % (Additional file 1: Table S1).

Relative abundances of the microbial community were also analyzed based on direct shotgun sequence (DSS) contigs. Major genera identified using this method were designated genomic information-rich genera, because contig information was used in analyses of metabolic ability. Nine genomic information-rich genera were identified, namely, *Hydrogenobaculum, Vulcanisaeta, Thermoproteus, Caldisphaera, Sulfolobus, Caldivirga, Acidithiobacillus*, *Thiomonas,* and *Metallosphaera* (Table 2 and Additional file 1: Figure S1). It was noteworthy that the order of the ranking between the two lists, the information-rich genera and the 20 abundant genera from 16S rRNA gene-based method, were similar, with *Hydrogenobaculum* at the top of both lists and all genomic information-rich genera in the top 20 of the 16S rRNA gene-based diversity survey.

**Table 2:** Information-rich genera and the top 20 most abundant genera derived from SNP hot springs water

Rank in abundance	Information-rich genera^a^		Top 20 most abundant genera identified by 16S rDNA analysis	
1	***Hydrogenobaculum***	5.73 %	***Hydrogenobaculum***	86.31 %
2	***Vulcanisaeta***	3.64 %	*Nanoarchaeum*	0.99 %
3	***Thermoproteus***	2.82 %	***Acidithiobacillus***	0.85 %
4	***Caldisphaera***	2.66 %	***Thermoproteus***	0.67 %
5	***Sulfolobus***	2.43 %	***Caldisphaera***	0.47 %
6	***Caldivirga***	2.39 %	***Thiomonas***	0.26 %
7	***Acidithiobacillus***	2.17 %	*Acidicaldus*	0.23 %
8	***Thiomonas***	1.66 %	*Sulfurisphaera*	0.21 %
9	***Metallosphaera***	1.50 %	*Acidianus*	0.17 %
10			***Caldivirga***	0.16 %
11			***Metallosphaera***	0.16 %
12			***Vulcanisaeta***	0.16 %
13			*Thiobacillus*	0.14 %
14			***Sulfolobus***	0.12 %
15			*Stygiolobus*	0.11 %
16			*Thermocladium*	0.10 %
17			*Pyrobaculum*	0.05 %
18			*Desulfurella*	0.04 %
19			*Acidimicrobium*	0.02 %
20			*Propionibacterium*	0.02 %

^a^Genera contained the relative abundance of contigs exceeding 1 %

The name in bold represents the genus share between two lists

### Inconsistencies between compositional lists of 16S rRNA gene-based diversity and metagenomic information

Ranking and composition of dominant microbes differed between the genomic information-rich genera list and the dominant microbe list (derived from 16S rRNA gene-based diversity surveys) identified in the present study. There were several potential reasons, including variations among microbes in genome sizes and copy numbers of 16S rRNA gene, and the threshold used. For example, although *Nanoarchaeum* was one of the most abundant genera in the top 20 16S rRNA gene-based list, it was absent from the list of the genomic information-rich genera (Table 2). This was attributed to its small genome (~490 kb; [27]), which would reduce the probability of being detected during sequencing.

### Advantageous characteristics of *Hydrogenobaculum* in SHP

*Hydrogenobaculum* was the predominant genus in SHP, where the temperature and pH were 69 °C and 2.5, respectively. Similarly, bacteria of the same genus also predominated in other acidic hot springs with variable (albeit harsh) environmental conditions, including Dragon Spring (70 ~ 72 °C; pH 3.1; [25, 26]), One Hundred Spring (73 °C; pH 3.5; 25, 26) and Norris Geyser (65 °C; pH 3.0; [26]).

**Fig. 2:**
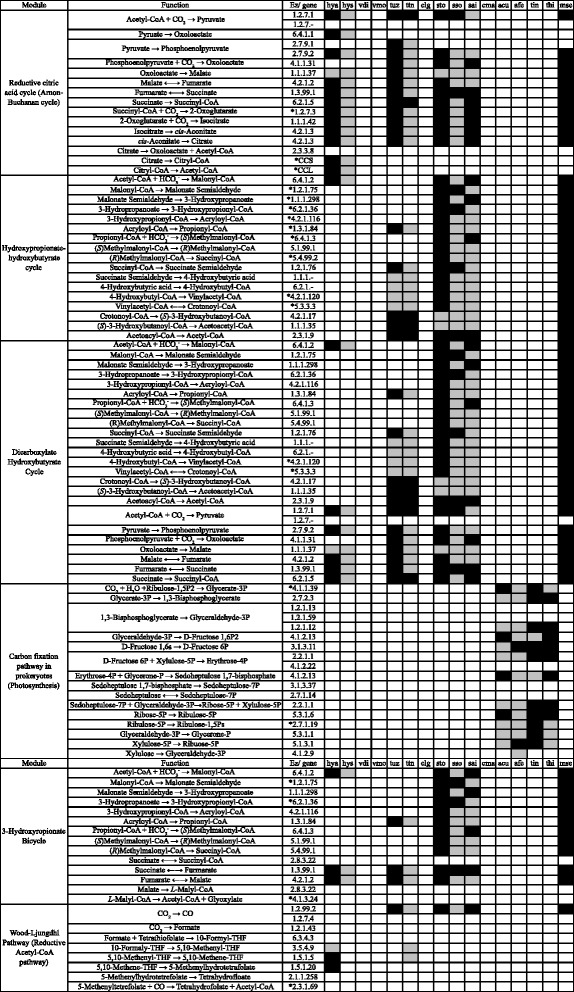
Carbon-metabolizing enzymes identified from dominant microbes using KEGG mapping. Asterisk: key enzymes in metabolic pathways. Abbreviations: hya, *Hydrogenobaculum* sp. Y04AAS1; hys, *Hydrogenobaculum* sp. SN; vdi, *Vulcanisaeta distributa*; vmo, *Vulcanisaeta moutnovskia*; tuz, *Thermoproteus uzoniensis*; ttn, *Thermoproteus tenax*; clg, *Caldisphaera lagunensis*; sto, *Sulfolobus tokodaii*; sso, *Sulfolobus solfataricus*; sai, *Sulfolobus acidocaldarius*; cma, *Caldivirga maquilingensis*; acu, *Acidithiobacillus caldus*; afe, *Acidithiobacillus ferrooxidans*; tin, *Thiomonas arsenitoxydan*; thi, *Thiomonas intermedia*; mse, *Metallosphaera sedula*. Cell in white, not listed in the KEGG reference pathway; grey, listed in the KEGG reference pathway; black, listed in the KEGG reference pathway and identified in our metagenomic dataset. Carbon-metabolizing enzymes identified from dominant microbes using KEGG mapping

The abilities of *Hydrogenobaculum* bacteria to assimilate carbon and metabolize hydrogen were suggested as crucial characteristics for living in an acidic hot spring [28, 29]. Indeed, carbon assimilation ability would be important for bacteria residing in SHP, due to the low dissolved organic carbon (DOC) concentration (1 mg/L) in spring water. However, *Hydrogenobaculum* was not the only microbial genus in SHP that assimilated inorganic carbon. Based on our metagenomic analysis, genes for carbon assimilation pathways were present in five of the nine genomic information-rich genera, including *Acidithiobacillus*, *Hydrogenobaculum*, *Metallosphaera, Sulfolobus,* and *Thiomonas* (Fig. 2). Also, physiological studies indicated that *Thermoproteus tenax* [30]*, Sulfolobus tokodaii* [31]*, Acidithiobacillus* [32], and *Metallosphaera* [33] were also capable of utilizing hydrogen as an energy source.

Although carbon assimilation metabolism and hydrogen metabolism (Additional file 1: Table S2) were regarded as important, they were not the only advantageous characteristics enabling the genus *Hydrogenobaculum* to dominate in SHP. Given substantial environmental variations among various hot springs, *Hydrogenobaculum* bacteria seemed to adapt to a broader temperature range compared to other detected genera; this could be another characteristic contributing to their dominance in variable acidic hot springs, with temperatures ranging from 50 to 82 °C [6, 15, 18, 25, 26, 34]. In SHP, water temperature ranged from 50 to 85 °C in a-year-long survey [21], similar to the temperature range in other *Hydrogenobaculum*-dominated hot springs. On the contrary, two other relatively less well represented genera identified in SHP, e.g. *Acidithiobacillus* and *Thiomonas*, were reported to only grow under mild thermophilic conditions. For example, temperature ranges of *A. caldus*, *A. ferrooxidans, Thiomonas arsenitoxydans,* and *Thiomonas intermedia*, were 32 ~ 52 °C [35], 10 ~ 37 °C [36], 30 °C [37], and 30 ~ 35 °C [37], respectively (Additional file 1: Table S3). Whether those bacterial strains have evolved additional heat tolerance mechanisms is apparently unknown.

Low oxygen concentrations in SHP water could also have affected microbial dominance, as they might not have been favorable for aerobic carbon assimilators, e.g. *Sulfolobus* and *Metallosphaera* [31, 38–40]. However, the oxygen requirement of *Hydrogenobaculum* Y04AAS1-related strain has apparently not been reported. Regardless, Y04AAS1-related strain seemed well adapted to anaerobic or microaerobic conditions, due to the presence of oxygen-sensitive pyruvate synthase and phosphoenolpyruvate carboxylase, which catalyze carboxylation steps in the reductive citrate cycle [41]. In addition, based on previous metagenomic studies, *Hydrogenobaculum* bacteria dominated in two acidic hot springs with radically different dissolved oxygen concentrations (>3 and 22 μM in Dragon Spring and One Hundred Spring, respectively; [25, 26]), suggesting substantial physiological flexibility of *Hydrogenobaculum* bacteria to variations in oxygen concentration. In short, dominance of *Hydrogenobaculum* bacteria in SHP was attributed to their inherent adaptability to withstand fluctuations in both temperature and oxygen concentration, as well as their metabolic capacity to assimilate carbon or use hydrogen as an energy source.

### Genomic map of *Hydrogenobaculum* bacteria

**Fig. 3:**
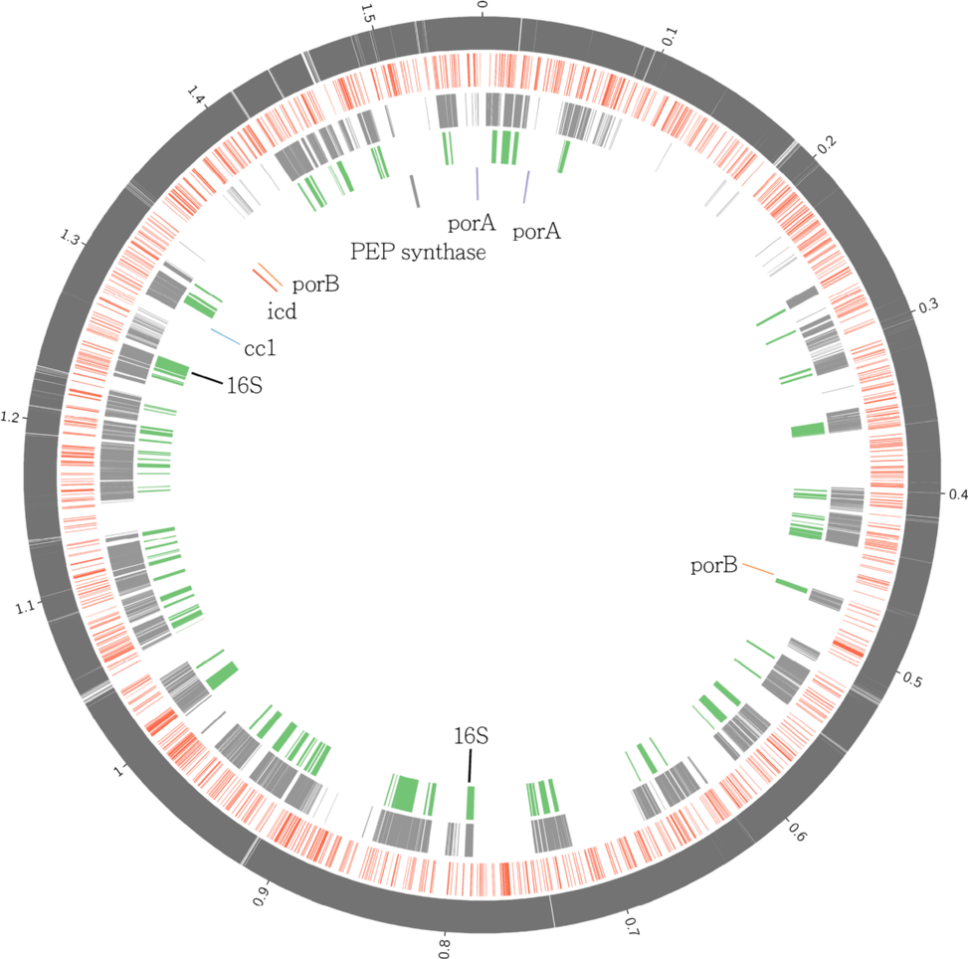
Mapping both fosmid and DSS contigs and raw reads to *Hydrogenobaculum* sp. Y04AAS1 reference genome. From outer to inter circles are: DSS raw reads (*dark gray*), DSS contigs (*orange*), fosmid raw reads (*light gray*), fosmid contigs (*green*) and specific highlighted genes, respectively

*Hydrogenobaculum* was the predominant genus in SHP. Mapping DSS reads covered >90 % of the length of the *Hydrogenobaculum* sp. Y04SSA1 reference genome (Fig. 3, Additional file 1: Table S4), consistent with analysis of genomic information-rich genera, which designated *Hydrogenobaculum* Y04SSA1-related strain as the dominant microbe (Additional file 1: Table S3). In addition, 16S rRNA genes and key carbon metabolic gene *ccl*, which encodes citryl-CoA lyase, in the genome of Y04SSA1-related strain were also on the genome map (Fig. 3).

### Comparison among acidic hot spring metagenomes

**Fig. 4:**
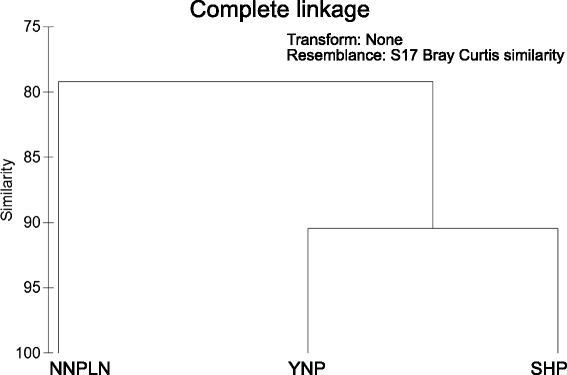
Cluster analysis of acidic hot spring metagenomes. Based on COGs clustering, YNP and SHP were more similar to each other than NNPLN. Nonetheless, the functional profiles of the three metagenomes shared a high similarity (>80 %). The clustering result based on KEGG pathways was also consistent with this clustering result (Additional file 1: Figure S6). YNP, SHP, and NNPLN were metagenomes of Yellowstone National Park (USA), Shi-Huang-Ping (Taiwan) and National Natural Park Los Nevados (Colombia), respectively

Cheng et al. reported that *Hydrogenobaculum* was a major genus in an acidic hot spring (Huang-Shan: 82.9 °C, pH 2.2) in TVG, based on amplification and analysis of full-length 16S rRNA genes [24]. *Hydrogenobaculum* was also designated the major genus in acidic hot springs in Yellowstone National Park [25, 26]. Comparative metagenomics characterize interactions between microbes and their environment. Currently, there are only two published acidic hot spring metagenome datasets [17, 18], one from Yellowstone National Park and the other from El Coquito spring, National Natural Park Los Nevados. Functional profiles (based on KEGG or COGs) of the SHP metagenome were compared to metagenomes of Yellowstone National Park and National Natural Park Los Nevados (for the latter, see Fig. 4 and Additional file 1: Figure S2). The SHP metagenome was closer to the metagenome from Yellowstone National Park than to National Natural Park Los Nevados. The four major pathways of the COGs category that differed between SHP/Yellowstone National Park and National Natural Park Los Nevados were: (a) amino acid transport and metabolism; (b) nucleotide transport and metabolism; (c) replication, recombination and repair; and (d) general function prediction (Additional file 1: Fig. S2).

Environmental conditions shape microbial community structure, which would in turn affect functional profiles. At SHP and YNP, conditions were: temperatures >50 °C, pH approximately 2–3, concentrations of sulfur-related compounds were high, and major microbial genera were *Hydrogenobaculum*, *Sulfolobus* and *Metallosphaera*. That these two hot springs were on distant continents and derived by distinct geological events, we concluded that microbial communities in acidic hot springs have undergone persistent and common selection, characterized by phenotypic conservation (Additional file 1: Fig. S2).

### Diverse microenvironments in SHP implied by microbial composition

**Fig. 5:**
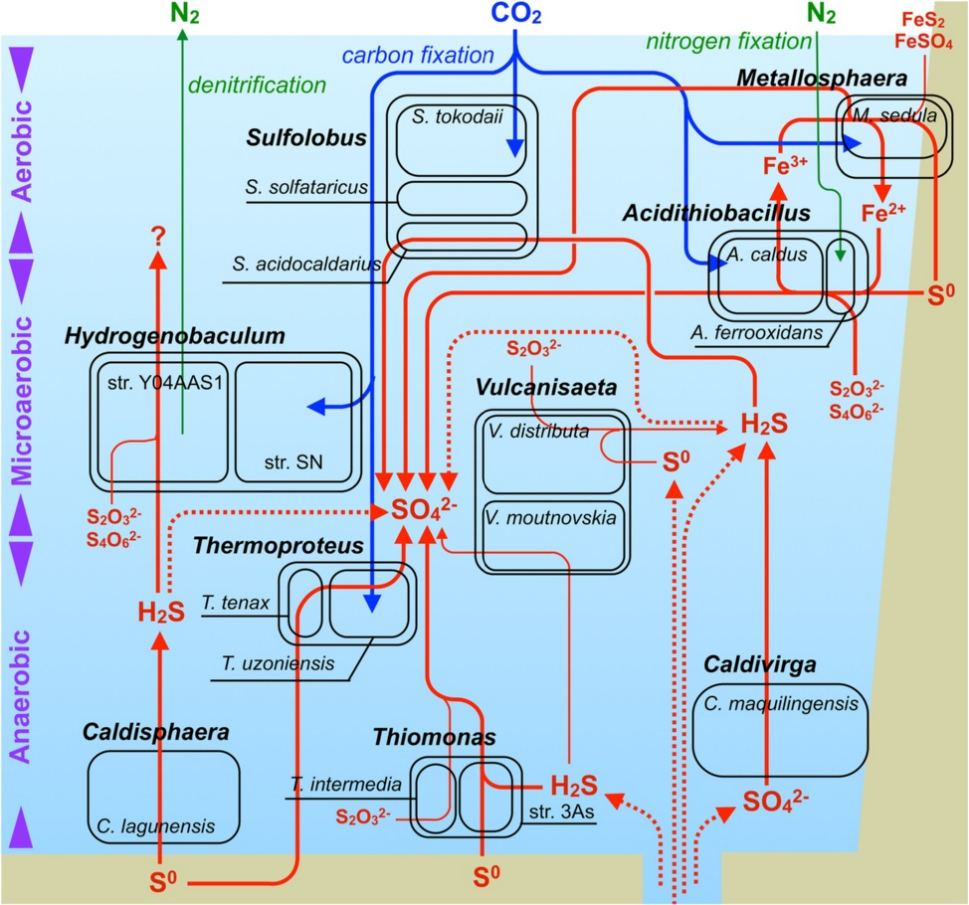
Hypothetical metabolic interactions between microbes and environments in the SHP acidic hot spring. These are potential relationships between dominant microbes and biogeochemical pathways of carbon, nitrogen and sulfur in the SHP acidic hot spring. Dominant microbial genera, species or strains are in rectangles. Dotted lines show compounds potentially derived from other sources (e.g., sediments, hot spring from underground, etc.). Thick lines are metabolic potentials detected in genera, whereas thin lines highlight alternative metabolic pathways in dominant microbial genera

To further elucidate interactions between microbes and environmental factors in SHP, we critically reviewed previous reports of dominant microbes in SHP. Analyzing microbial community structures and metagenomes contribute to understanding geochemical conditions in acidic hot springs [42]. Dominant microbes in SHP microbial community had diverse oxygen preferences, including aerobic microbes (e.g., *Sulfolobus* and *Metallosphaera*), facultative aerobic microbes (e.g., *Acidithiobacillus*), microaerobic microbes (e.g., *Vulcanisaeta* and *Caldvirga*), and anaerobic microbes (e.g., *Thiomonas* and *Caldisphaera*). We inferred that the water environment of the hot spring had at least three distinct microhabitats, namely aerobic, microaerobic and anaerobic (Fig. 5 and Additional file 1: Table S3). Furthermore, the lowest reported oxygen condition in SHP (2.74 mg/L; [43]) indirectly supported the presence of habitats with varying oxygen concentrations.

Although metagenomic information in the present study clearly supported the presence of microaerobic or anaerobic microenvironments in SHP water, potential sources of error could not be excluded. For example, some microaerobic or anaerobic microbes from the sediment or the soil nearby the pond might have contaminated our sample. However, that pond water was clear and calm during sampling, and sampling was carefully conducted, the probability that contamination occurred was extremely low.

### Carbon cycle in the SHP

The carbon cycle in the acidic hot spring is highly dependent upon chemotrophic processing, as the combination of high temperature and low pH hamper photosynthesis [19, 44]. The upper limit for photosynthesis is ~ 56 °C in an acidic (pH <4.0) environment [44]. Thus, microbes detected in the springs of Cater Hills and Horris Greyser Basin (USA) presumably used non-photosynthetic chemotrophic pathways for carbon assimilation [16].

Four chemotrophic carbon assimilation pathways were identified in our metagenomic data. *Hydrogenobaculum* had a reductive citrate cycle, *Sulfolobus* and *Metallosphaera* used the hydroxypropionate-hydroxybutyrate cycle [45, 46], and *T. uzoniensis* and *T. tenax* had genes for both a reductive citrate cycle and a dicarboxylate-hydroxybutyrate cycle [47, 48]. We identified two chemosynthesis-based carbon assimilation pathways, including a reductive citrate cycle in genus *Hydrogenobaculum*, and a hydroxypropionate-hydroxybutyrate cycle in genus *Sulfolobus* and genus *Metallosphaera* (Fig. 2 and Additional file 1: Table S5).

Two key genes in the reductive citrate cycle, *korA* (EC 1.2.7.3) and *korB* (EC 1.2.7.3), encoding the α and β subunits of critical enzyme 2-oxoglutarate ferredoxin oxidoreductase, were identified in the metagenome data and assigned to *Hydrogenobaculum* and *T. uzoniensis*. Furthermore, based on metagenomic data, *Hydrogenobaculum* bacteria had a gene encoding citryl-CoA lyase (*ccl*), an enzyme capable of catalyzing a biochemical reaction similar to another essential enzyme, ATP-citrate lyase, in the reductive citrate cycle [49].

In addition to the reductive citrate cycle, both *T. uzoniensis* and *T. tenax* had the key enzyme 4-hydroxybutyryl-CoA dehydratase (4.2.1.120 and 5.3.3.3) for dicarboxylate-hydroxybutyrate, and the key enzyme 2-oxoglutarate synthase (KorA and KorB, EC 1.7.3.2) of the reductive citrate cycle.

*Sulfolobus* and *Metallosphaera* bacterial rely on the hydroxypropionate-hydroxybutyrate cycle to convert carbon dioxide into organic carbons. Genes encoding key enzymes in this pathway, including acetyl-CoA/propionyl-CoA carboxylase (EC 6.4.1.2), malonyl-CoA reductase (EC 1.2.1.75 and 1.1.1.298), methylmalonyl-CoA mutase (EC 6.4.99.2) and 4-hydroxybutyryl-CoA dehydratase (EC 4.2.1.120) [41] were identified in contigs assigned to *Sulfolobus tokodaii* and *Metallosphaera* genus.

*Acidithiobacillus* and *Thiomonas* bacteria use the Calvin cycle to assimilate inorganic carbon [50–52]. Notably, *Acidithiobacillus* bacteria use electrons generated from sulfur metabolism for the Calvin cycle [50, 51], thereby circumventing temperature limitations for photosynthesis [19]. However, it remains unclear whether *Thiomonas* bacteria could invoke a mechanism similar to *Acidithiobacillus* bacteria, enabling it to fix carbon [21] when the water temperature increased. However, the presence of *cbbSL* genes that encode the key enzyme ribulose 1,5-bisphosphate carboxylase/oxygenase (EC 4.1.1.39) in the Calvin cycle identified in the current study and a previous report (Fig. 2; [52]), provided additional evidence that *Thiomonas* bacteria can assimilate carbon.

### Nitrogen cycle in SHP

**Fig. 6:**
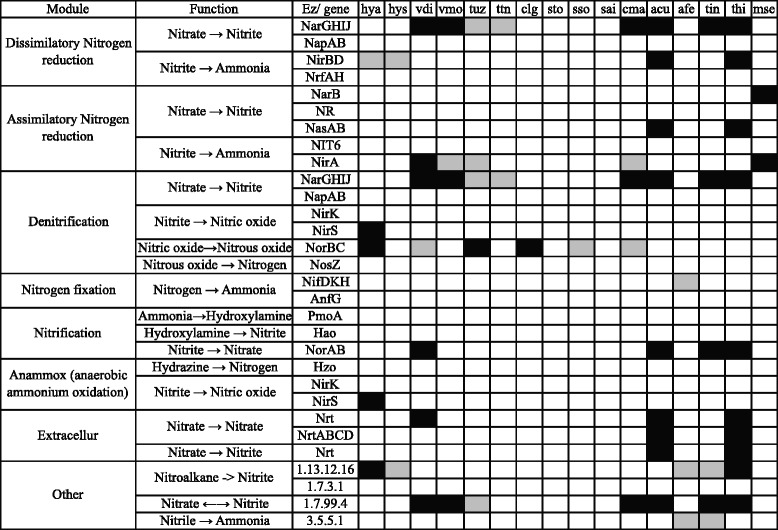
Nitrogen-metabolizing enzymes identified from dominant microbes using KEGG mapping. For detailed descriptions for abbreviations and color codes, please see the legend for Fig. 2

The only dominant SHP microbe capable of fixing nitrogen (Fig. 6, Additional file 1: Table S5; [53]) was *A. ferrooxidans*; therefore, we inferred it played a key role in the SHP nitrogen cycle. It is noteworthy that the SHP spring is a nitrogen-limited environment (nitrate concentrations ranged from 0.9 ppm to below detection limits; Table 1; [21]). In addition, microbes living in SHP might have to obtain organic nitrogen from an alternative source (e.g. metabolizing existing nitrogen-containing compounds in the water). For example, several bacteria (*Hydrogenobaculum*, *A. ferrooxidans*, and *Thiomonas*) had nitronate monooxygenase (EC 1.13.12.16), the enzyme for transforming nitroalkane compounds (R-NO_2_) to nitrite (Fig. 6).

Nitrite could be converted to ammonia (nitrogen reduction) and used to synthesize amino acids, or be converted into nitrogen (through denitrification) to generate energy. Two groups of bacteria, genus *Thiomonas* and *A. ferrooxidans* encoded several genes (*narG*, *narH*, *narI*, *narJ*, *nirB and nirD*) involved in dissimilatory nitrate reduction pathways. Nevertheless, according to the KEGG reference pathway, none of the dominant microbes had enzymes for a complete denitrification pathway (Fig. 6). Even though Reysenbach et al. analyzed the *Hydrogenobaculum* bacterial genome and suggested that str. Y04AAS1 genome harbored all genes required for this pathway, they did not detect reduced nitrate under experimental conditions [28]. However, that SHP has a low concentration of organic nitrogen compounds, microbes might prefer to use nitrate to synthesize building blocks in lieu of generating energy. Clearly, further investigations are needed to elucidate the nitrogen nutrient cycle in SHP.

### Sulfur metabolism

**Fig. 7:**
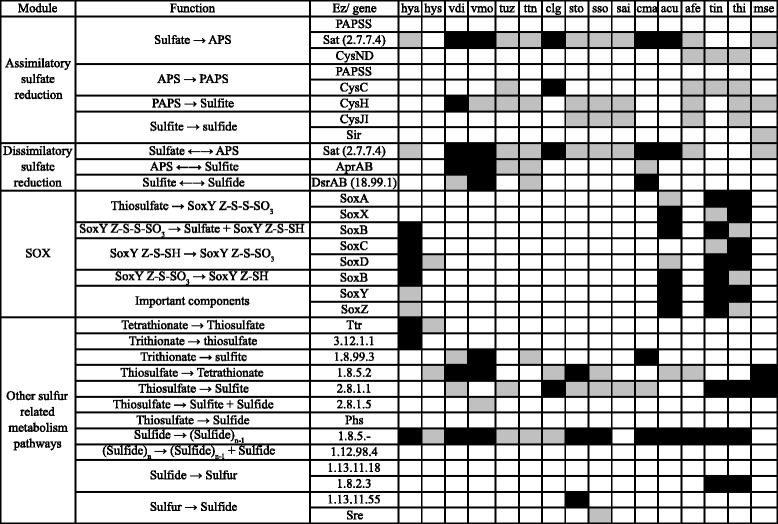
Sulfur-metabolizing enzymes identified from dominant microbes using KEGG mapping. For detailed descriptions for abbreviations and color codes, please see the legend for Fig. 2

Dominant microbes were dexterous in sulfur metabolism (Fig. 7). *Vulcanisaeta* archaea, *Thermoproteus tenax* and *Caldivirga maquilingensis* had the capacity to transform trithionate into sulfite with sulfite reductase (EC 1.8.99.3). Since archaea of genus *Vulcanisaeta*, *T. tenax* and *C. maquilingensis* had all enzymes required for dissimilatory sulfate reduction, they were capable of utilizing sulfate or sulfite for energy metabolism. Furthermore, sulfite converted from trithionate could be used for dissimilatory sulfate reduction. *Thiomonas* bacteria encoded genes for complete SOX complex, enabling them to convert thiosulfate into sulfate. Thiosulfate could also be converted into sulfite via thiosulfate/3-mercaptopyruvate sulfurtransferase (EC 2.8.1.1), present in most dominant microbes in SHP. Although several dominant microbes could convert thiosulfate into tetrathiosulfate, *Hydrogenobaculum* bacteria were the only dominant microbes capable of converting either tetrathionate or trithionate into thiosulfate.

Searching against the KEGG database provided a basic understanding of the SHP sulfur cycle in the SHP. However, an extended literature search revealed additional or uncommon sulfur-related metabolic pathways, absent from the KEGG reference pathways, but identified in dominant microbes. For example, in addition to genus *Hydrogenobaculum*, based on genomic and transcriptomic analyses, we inferred that *S. tokodaii* [54], genus *Acidithiobacillus* [50, 55, 56] and genus *Metallosphaera* [54] could also convert tetrathionate to thiosulfate (Additional file 1: Figure S3, Table S5). For bioleaching microbes like genus *Acidithiobacillus* and genus *Metallosphaera*, thiosulfate served as an oxidizer for Fe(II), which could be used to generate protons as a driving force for respiration [54, 57]. Polysulfide mechanism is another Fe(II) oxidizing pathway [55, 57]. Interestingly, based on the KEGG reference pathway and a literature search, almost all dominant microbes (including non-bioleaching microbes) in SHP were capable of transforming hydrogen sulfide to polysulfide (Additional file 1: Figure S3, Table S5). Regardless, *T. tenax* and *A. caldus* were the only two dominant microbes with enzymes to recycle polysulfide [48, 50] and thereby replenish the hydrogen sulfide pool, which would be beneficial for *A. caldus* during bioleaching.

### Hydrogen metabolism

Genus *Hydrogenobaculum* could use hydrogen as its major energy source [29]. To explore hydrogen metabolism-related genes in our metagenomics data, we searched our DDS dataset against the NCBI database, and summarized the results (Additional file 1: Table S2). The gene encoding Ni/Fe hydrogenase, which catalyzes the reaction: H_2_ ↔ 2H^+^ + 2e^−^, was identified. In addition, genes encoding Hyp, a group of proteins required during maturation of Ni/Fe hydrogenase [58], were also present in our DSS dataset.

### Microbial interactions in acidic hot springs

In addition to several potential metabolic interactions, 16S rRNA gene-based diversity and CRISPR arrays also revealed microbe-microbe interactions. In that regard, the presence of genus *Nanoarchaea* and numerous viral sequences/CRISPR arrays were consistent with robust microbial interactions in the SHP.

Genus *Nanoarchaea* (represented by *Nanoarchaea*-like 16S rRNA gene sequences) was a dominant genus in SHP (Fig. 1 and Table 2). There were apparently no previous reports of genus *Nanoarchaea* in an acidic thermal environment with a low NaCl concentration. The sole species of genus *Nanoarchaea* (*Nanoarchaeum equitans*) previously reported had a much-reduced genome and could only be grown in the presence of *Ignicoccus sp.*, an archaeal genus [59]. Furthermore, that an *Ignicoccus*-like 16S rRNA gene sequence was also detected in the present survey (highlighted in green in Additional file 1: Table S1), suggested a potential host-parasite interaction between *Nanoarchaea* and *Ignicoccus*.

It is well known that CRISPR is an antiviral defense system common in microbial genomes [60, 61]. Furthermore, repeat sequences and spacers in CRISPR assays can be used to assign taxa, as they are strain-specific [62]. In the SHP metagenome, 1711 CRISPR-like arrays (comprising 15130 spacers) were identified, of which 123 were assigned to specific microbes (based on their unique repeat sequences; Additional file 1: Table S6). In addition, there were several kinds of viral DNA sequences in the SHP metagenome (Additional file 1: Table S7), providing evidence of viral infection.

**Fig. 8:**
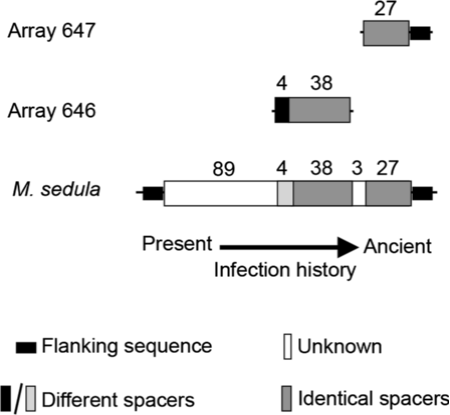
Alignment of CRISPR arrays with *M. sedula* reference arrays. Numbers of spacer are denoted. According to the CRISPR database, *M. sedula* has four CRISPR arrays in its genome. The repeat sequence of Array 647 and 646 (detected in the SHP metagenome by BLAST) matched repeat sequences of the longest CRISPR array (CRISPR ID: NC_009440_4, refer as “reference array” hereafter) in the *M. sedula* DSM 5348 reference genome. Since the order of spacers could be associated with time of virus infection, older spacers were located near the “ancient” end of the array. With a comparative analysis of the spacer sequences, all 27 spacers in Array 647 perfectly matched the array in the reference genome (with regards to sequence similarity and orientation). The 27 spacers were located at the end of the reference array. Array 646 contained 42 spacers; the last 38 spacers perfectly matched the 83^th^ to 121^st^ spacers in the reference array. Sequences of the first four spacers in Array 646 were different from all of the spacers in reference array; therefore, there was divergence of virus infection history between the *M. sedula*-related species in the SHP and *M. sedula* DSM5348. Moreover, since no spacers similar to the 1^st^ to 89^th^ and 122^nd^ to 124^th^ spacers of the reference array were detected, those spacers were designated “unknown”

Spacer sequences of the CRISPR array could be used to characterize microbial evolution. Six of the CRISPR-like arrays identified from DSS dataset were assigned to *Metallosphaera sedula* based on their repeat sequence*.* The *M. sedula* reference genome contained four CRISPR arrays, each with a unique repeat sequence. Six CRISPR-like arrays were compared to known CRISPR arrays in the *M. sedula* reference genome [63]; two of the CRISPR-like arrays had identical repeat sequences with that of the longest CRISPR array (161 spacers) from the *M. sedula* reference genome. Furthermore, there were 65 identical spacers identified by comparing spacer sequences of those two arrays to the reference array (Fig. 8). More importantly, identical spacers were arranged in the same order as the reference. Since spacers are added to a CRISPR array in a chronological order [64], with 65 identical spacers in the reference genome on the 3′-end, we inferred that the two *M. sedula* populations, the reference strain isolated in Italy, and another identified by analyzing metagenomic data from SHP in this study, were both derived from the same ancestral population (with a common infection history). The *M. sedula* type strain was isolated from a hot water pond at Pisciarelli Solfatara, Italty. Multiple water samples were collected for microbial isolation, water pH was ~ 2 and temperature ranged from 25 to 52 °C [40] (cooler than SHP).

## Conclusions

Using a metagenomic approach to acquire copious sequence data from members of SHP planktonic microbial community enabled us to not only identify community composition, but also to postulate potential interactions within the ecosystem. Specifically, we used metagenomic data to predict potential metabolite exchange, microbe-phage interaction (CRISPR analyses) and archaeal parasite-host interactions (*Nanoarchaea* and *Ignicoccus*) within SHP. Potential metabolite exchanges among microbes is shown (Fig. 8), based on existing physiological and biochemical studies of dominant microbes. Predicting potential metabolic pathways for carbon, sulfur, carbon and hydrogen with the NCBI and/or KEGG databases enabled us to elucidate metabolic ability of each dominant microbe. However, metabolic analyses cannot fully explain the *Hydrogenobaculum*-dominant feature. Previous studies attributed a *Hydrogenobaculum*-dominant feature based on carbon assimilation pathways and hydrogen utilization features of this genus. However, *Hydrogenobaculum* is not the only microbial genus capable of utilizing hydrogen and assimilating inorganic carbon. Thus, we proposed that *Hydrogenobaculum* bacteria dominate SHP due to additional abilities, e.g. temperature tolerance and ability to survive in an anaerobic environment. Together with our analytical results and literature mining, this study provided a comprehensive understanding of interactions within the microbial ecosystem in an acidic thermal environment.

## Methods

### Sample collection and preparation

For direct shotgun sequencing (DSS), water samples were collected from an SHP hot spring (25°11′43. 60″N, 121°36′8. 82″E; water depth was about 15 ~ 20 cm at sampling site) into sterile polypropylene (PP) containers on April 25^th^, 2012. Water temperature and pH were measured *in situ*; all environmental parameters listed in Table 1 were measured according to National Institute of Environmental Analysis (NIEA, Taiwan) or American Public Health Associate (APHA) protocols. A tangential flow system [65] equipped with hollow-fiber cartridge (pore diameter 0.2 μm; Hollow Fiber Cartridge CFP-2-E-3MA, GE Healthcare, Little Chalfont, United Kingdom) was used to reduce the volume of spring water to approximately 500 mL. Thereafter, the water sample was subjected to high-speed centrifugation (7000 × *g* for 30 min; Himac CR-21, Hitachi, Japan). Sampled water was stored at 4 °C before further processing. Ample supernatant was retained for re-suspension of pellets into a muddy solution that yielded a mixture of suspension particles, fine sediments and microorganisms. Thereafter, DNA was extracted with an UltraClean® Mega Soil DNA Isolation Kit, (MO BIO Laboratories, Inc., Carlsbad, CA, USA), according to the manufacturer’s protocol.

For fosmid library construction, water samples were collected on December 3^rd^, 10^th^ and 21^st^ 2010, and on February 8^th^ and March 17^th^, 2011 at the same location, and processed as described above.

### Fosmid library construction

Fosmid libraries were constructed according to manufacturer’s instructions (Copy Control™ HTP Fosmid Library Production Kits, Epicentre® Biotechnologies, Madison, WI, USA). Pulse-field gel electrophoresis (PFGE) was done to verify insert sizes of fosmid DNA from randomly selected colonies. Overnight cultures of selected colonies were harvested by centrifugation (7000 × *g* at 4 °C for 30 min; Himac CR-21, Hitachi, Japan). Fosmid DNA was extracted according to manufacturer’s instructions (QIAGEN Plasmid Mini Kit, QIAGEN, Venlo, The Netherlands). Insert DNA was removed with a restriction enzyme (*Not*I) at 37 °C for 16–18 h. For PFGE, digested DNA was analyzed using 1 % agarose gel in 1/3 × Loening Buffer. The PFGE system consisted of a Standard Power Pack (Rotaphor® System, Biometra, Goettingen, Germany) and a circulator tank (Refrigerated Circulator RCB411, TKS, Kaiserslautern, Germany) for cooling. After confirmation of insert size, a fosmid library MG-HSTL (9481 clones) was constructed and deposited in the Food Industry Research and Development Institute (FIRDI, Taiwan; publicly available as of Aug 1^st^, 2013. Website: http://www.firdi.org.tw/En_Firdi_Index.ASPX).

Fosmid DNA was extracted from randomly picked 1485 clones according to the alkaline lysis method, provided by VYM Genome Research Center, National Yang-Ming University, Taiwan. Concentration of fosmid DNA was measured using a Qubit Fluorometer (Life Technologies, Carlsbad, CA, USA) and then mixed (equal amounts of DNA from each clone) into a bulk sample for sequencing.

### Random shotgun sequencing and contig assembly

Metagenomic DNA was directly extracted from the water sample and sequenced on a HiSeq™ 2000 (Illumina, San Diego, CA, USA) at Yourgene Bioscience Co., Ltd. (Taipei, Taiwan). Metagenomic DNA was sequenced separately from the Fosmid library. Raw sequencing reads were trimmed (35 bp minimum length and error probability <0.05). All DSS and fosmid contigs were assembled by MetaVelvet [66]. Raw sequence data to be assembled were ~ 56.3 and 22.4 Gb for DSS and the fosmid library, respectively. Statistics regarding sequencing and contig assembling for DSS and fosmid are shown (Additional file 1: Tables S8 and S9, respectively).

Sequence information from DSS was used to determine composition and metabolic potential of microbial community, and reconstruct the *Hydrogenobaculum* bacterial genome. In addition, sequence information obtained from the fosmid library was used to facilitate reconstruction of the *Hydrogenobaculum* bacterial genome.

### Analysis of microbial community structure

Microbial community structure in SHP was characterized by 16S rRNA gene-based diversity surveys. Qualified reads were blasted against the SILVA SSU reference database (Version 115, download date: Sep 7^th^, 2013) with the following criteria: a) sequence identity >95 %; b) alignment coverage >90 % of the length of the query sequence; c) E-value <10^−15^; and d) highest bit-score. The taxonomic affiliation of top hit in the blast search was assigned for a single read. The relative abundance (*RA*_*16S*_) of a specific genus was calculated by the total read number in a genus, divided by the total 16S rRNA gene sequence encoded reads (Additional file 1: Table S1). The top 20 abundant genera were selected for phylogenetic analyses (Fig. 1), using web-based software (GraPhlAn; http://huttenhower.org/galaxy, [67]).

### Relative abundance of genomic information-rich genera

Each contig was assigned to a specific organism by blasting it (E-value ≤10^−5^) against the NCBI database. Taxa of the contigs were determined by annotation of the best hit. Relative abundance (*RA*_*contig*_) of each genus was calculated as total number of qualified reads in contigs belonging to the same genus, divided by total number of qualified reads. Genera with a *RA*_*contig*_ >1 % (Additional file 1: Figure S1), were designated as genomic information-rich genera. Furthermore, to present diverse metabolic capabilities within the same genera, metagenomic information or relevant literature for major strains or species listed under genomic information-rich genera with *RA*_*contig*_ >0.2 % were also retrieved (Additional file 1: Table S3). Two lists of dominant microbial genera were generated: a) top 20, the most abundant genera (based on 16S rRNA gene-based diversity surveys); and b) genomic information-rich genera (based on taxonomic affiliations of contigs) in SHP, according to relative abundance analyses.

### Mapping contigs onto *Hydrogenobaculum* bacterial genome

The majority of the DNA recovered was from the genus *Hydrogenobaculum* (see Results). Therefore, the genome of *Hydrogenobaculum sp.* Y04AAS1 [28], downloaded from Joint Genome Institute (JGI), was used as a reference genome for contig mapping. Qualified sequencing reads of DSS and fosmid were mapped to the reference genome by CLC Genomics Workbench (similarity 0.7, mapping length 0.9, website: http://www.clcbio.com), whereas DSS and fosmid contigs were mapped by MUMmer 3.0, using default settings (website: http://mummer.sourceforge.net, [68]). Mapping results were visualized with Circos [69].

### Comparative metagenomics analysis

To compare putative functional profiles between the metagenome of this study and those of the other two acidic hot spring metagenomes from the Americas, two metagenomic datasets, namely Yellowstone National Park (#41119) and National Natural Park Los Nevados (#4449206.3), were downloaded from the NCBI and MG-RAST servers, respectively. Thereafter, all open reading frames retrieved from the three metagenomes were compared against COGs and KEGG databases using the WebMGA service (website: http://weizhong-lab.ucsd.edu/metagenomic-analysis/) and the BBH-method service in KAAS (website: http://www.genome.jp/kegg/kaas/), respectively. Normalization to samples was done by the matched ORFs in each category, divided by the total matched ORFs for each sample. Relative abundance was presented with a line plot (non-prokaryotic functions or pathways were excluded). Furthermore, Primer 6 (website: http://www.primer-e.com/primer.htm) was used to compare putative functional profiles of three metagenomes, using a Bray-Curtis model with complete-linkage cluster [70].

### Reconstruction of potential metabolic networks of dominant genera

The KEGG reference pathway mapping and blastp (E-value <10^−5^ and bit-score >100) were used to identify proteins associated with dominant microbes from DSS contigs. With KEGG Mapper (http://www.genome.jp/kegg/tool/map_pathway2.html), proteins involved in carbon (Fig. 2), nitrogen (Fig. 6), or sulfur (Fig. 7) metabolic pathways were identified by mapping gi codes obtained in blastp to KEGG reference pathways (Release 72.0, October 1^st^ 2014) for each dominant microbe. However, the KEGG database per se was regarded as insufficient as a reference for all acquired metagenomic information from an extreme microbial ecosystem [18, 25, 26]. Therefore, in addition to KEGG reference pathway mapping, information extracted from an extensive literature search was also used to predict metabolic networks and potential relationships among dominant microbes. Thereafter, relevant physiological, biochemical and genomic reports (Additional file 1: Table S3) were reviewed to provide additional information regarding carbon, sulfur and nitrogen sources (Additional file 1: Figure S3).

### Identification of CRISPR-like arrays

The PILER-CR software (website: http://drive5.com/piler/) was used to identify CRISPR-like arrays in our DSS dataset [71]. Repeats and spacers of identified CRISPR-like arrays were searched against CRISPRdb (website: http://crispr.u-psud.fr/crispr/; [63]) with blastN-short, E-value <10^−5^, to assign arrays to specific microbial species and to identify correlated viral sequences.

### Data deposition

The microbial metagenome elucidated in this study was deposited in the NCBI Sequence Read Archive (Accession Number SRP041649).

## Availability of supporting data

The microbial metagenome elucidated in this study was deposited in the NCBI Sequence Read Archive (Accession Number SRP041649).

## Additional file

Additional file 1:
**Supplimentary material on metagemic analysis.** (DOCX 312 kb)

## Abbreviations

CRISPR: Clustered regularly interspaced short palindromic repeats
DSS: Direct shot-gun sequencing
SHP: Shi-Huang-Ping
