# Supplementary material for: Metabolic characteristics of dominant microbes and key rare species from an acidic hot spring in Taiwan revealed by metagenomics
Source: BMC Genomics. 2015 Dec 3;16:1029. doi: 10.1186/s12864-015-2230-9 (PMC4668684; doi:10.1186/s12864-015-2230-9)
Supplement: Additional file 1: — Supplimentary material on metagemic analysis. (DOCX 312 kb) [file 12864_2015_2230_MOESM1_ESM.docx]

**SUPPLEMENTAL MATERIALS**

**Identify *Hydrogenobaculum* genes for hydrogen metabolism**

Hydrogen metabolism of extreme microbes has yet to be included in the KEGG database. However, *Hydrogenobaculum* sp. is known to have hydrogen metabolic ability. To investigate hydrogen metabolic ability of *Hydrogenobaculum* sp. Y04AAS1, the NCBI gene database was searched using keyword “hydrogenase”, and cross-referenced with the gene list under *Hydrogenobaculum* sp. or Y04AAS1. Results were summarized (Table S2).

**Mapping DSS contigs to *Hydrogenobaculum* and *E. coli* genomes**

To reconstruct the *Hydrogenobaculum* genome, *Hydrogenobaculum* sp. Y04AAS1 and *E. coli* DH10B reference genomes were downloaded from JGI (GenBank accession ID were CP001130 and CP000948.1 respectively). The DSS raw reads were mapped to *Hydrogenobaculum* sp. Y04AAS1 or *E. coli* DH10B reference genome with CLC Genomics Workbench (CLC Bio, Aarhus, Denmark). Similarity and length fraction settings used were listed (Table S4).

**Table S1. Microbial genera in SHP hot spring identified by analyzing 16S rRNA gene-encoded sequencing reads (16S rRNA-based diversity survey).**

| Rank | Genus | *RA_16S_* (%) | Rank | Genus | *RA_16S_* (%) | Rank | Genus | *RA_16S_* (%) |
| --- | --- | --- | --- | --- | --- | --- | --- | --- |
| 1 | *Hydrogenobaculum* | 86.31 | 58 | *Cupriavidus* | >0.01 | 115 | *Pseudomonas* | >0.01 |
| 2 | *Nanoarchaeum* | 0.99 | 59 | *Sulfuricella* | >0.01 | 116 | *Rhodanobacter* | >0.01 |
| 3 | *Acidithiobacillus* | 0.85 | 60 | *Alicyclobacillus* | >0.01 | 117 | *Salmonella* | >0.01 |
| 4 | *Thermoproteus* | 0.67 | 61 | *Paucimonas* | >0.01 | 118 | *Sideroxydans* | >0.01 |
| 5 | *Caldisphaera* | 0.47 | 62 | *Sediminibacterium* | >0.01 | 119 | *Thauera* | >0.01 |
| 6 | *Thiomonas* | 0.26 | 63 | *Acidocella* | >0.01 | 120 | *Uliginosibacterium* | >0.01 |
| 7 | *Acidicaldus* | 0.23 | 64 | *Legionella* | >0.01 | 121 | *Acetobacter* | >0.01 |
| 8 | *Sulfurisphaera* | 0.21 | 65 | *Ottowia* | >0.01 | 122 | *Achromobacter* | >0.01 |
| 9 | *Acidianus* | 0.17 | 66 | *Thermosphaera* | >0.01 | 123 | *Acidisphaera* | >0.01 |
| 10 | *Caldivirga* | 0.16 | 67 | *Woodsholea* | >0.01 | 124 | *Acidobacterium* | >0.01 |
| 11 | *Metallosphaera* | 0.16 | 68 | *Herbaspirillum* | >0.01 | 125 | *Acidovorax* | >0.01 |
| 12 | *Vulcanisaeta* | 0.16 | 69 | *Thiobacter* | >0.01 | 126 | *Aquicella* | >0.01 |
| 13 | *Thiobacillus* | 0.14 | 70 | *Bradyrhizobium* | >0.01 | 127 | *Arhodomonas* | >0.01 |
| 14 | *Sulfolobus* | 0.12 | 71 | *Caldimonas* | >0.01 | 128 | *Asticcacaulis* | >0.01 |
| 15 | *Stygiolobus* | 0.11 | 72 | *Limnobacter* | >0.01 | 129 | *Belnapia* | >0.01 |
| 16 | *Thermocladium* | 0.10 | 73 | *Thermofilum* | >0.01 | 130 | C1-B045 | >0.01 |
| 17 | *Pyrobaculum* | 0.05 | 74 | *Acidiferrobacter* | >0.01 | 131 | CL500-29 marine group | >0.01 |
| 18 | *Desulfurella* | 0.04 | 75 | *Coxiella* | >0.01 | 132 | *Chroococcidiopsis* | >0.01 |
| 19 | *Acidimicrobium* | 0.02 | 76 | *Sulfophobococcus* | >0.01 | 133 | *Corynebacterium* | >0.01 |
| 20 | *Propionibacterium* | 0.02 | 77 | *Thermocrinis* | >0.01 | 134 | *Cytophaga* | >0.01 |
| 21 | *Craurococcus* | 0.02 | 78 | *Thermodiscus* | >0.01 | 135 | *Dechloromonas* | >0.01 |
| 22 | *Defluviicoccus* | 0.02 | 79 | *Acidiplasma* | >0.01 | 136 | *Desulfomonile* | >0.01 |
| 23 | *Acidiphilium* | 0.02 | 80 | *Azoarcus* | >0.01 | 137 | *Desulfonauticus* | >0.01 |
| 24 | *Acidilobus* | 0.02 | 81 | *Enterobacter* | >0.01 | 138 | *Desulfovermiculus* | >0.01 |
| 25 | *Thermoplasma* | 0.02 | 82 | *Hydrotalea* | >0.01 | 139 | *Diaphorobacter* | >0.01 |
| 26 | *Flavobacterium* | 0.01 | 83 | *Ignisphaera* | >0.01 | 140 | *Dokdonella* | >0.01 |
| 27 | *Ferritrophicum* | >0.01 | 84 | *Nevskia* | >0.01 | 141 | *Erwinia* | >0.01 |
| 28 | *Sulfobacillus* | >0.01 | 85 | *Picrophilus* | >0.01 | 142 | *Erythrobacter* | >0.01 |
| 29 | *Zoogloea* | >0.01 | 86 | *Undibacterium* | >0.01 | 143 | *Ferrithrix* | >0.01 |
| 30 | *Gluconacetobacter* | >0.01 | 87 | *Variovorax* | >0.01 | 144 | *Iamia* | >0.01 |
| 31 | *Pyrodictium* | >0.01 | 88 | *Aquifex* | >0.01 | 145 | *Klebsiella* | >0.01 |
| 32 | *Sulfuritalea* | >0.01 | 89 | *Ferruginibacter* | >0.01 | 146 | *Leptospirillum* | >0.01 |
| 33 | *Nitrosomonas* | >0.01 | 90 | *Fervidicoccus* | >0.01 | 147 | *Mariprofundus* | >0.01 |
| 34 | *Desulfurococcus* | >0.01 | 91 | *Flavisolibacter* | >0.01 | 148 | *Methylobacillus* | >0.01 |
| 35 | *Methylibium* | >0.01 | 92 | *Granulosicoccus* | >0.01 | 149 | *Methylophaga* | >0.01 |
| 36 | *Halorhodospira* | >0.01 | 93 | *Metallibacterium* | >0.01 | 150 | *Methylophilus* | >0.01 |
| 37 | *Lautropia* | >0.01 | 94 | *Paenibacillus* | >0.01 | 151 | *Nitrosococcus* | >0.01 |
| 38 | *Nitrococcus* | >0.01 | 95 | *Pusillimonas* | >0.01 | 152 | *Novosphingobium* | >0.01 |
| 39 | *Nitrosospira* | >0.01 | 96 | *Rhodocyclus* | >0.01 | 153 | *Pantoea* | >0.01 |
| 40 | OM43 clade | >0.01 | 97 | *Rhodovarius* | >0.01 | 154 | *Phenylobacterium* | >0.01 |
| 41 | *Hydrogenobacter* | >0.01 | 98 | *Achromatium* | >0.01 | 155 | *Propionivibrio* | >0.01 |
| 42 | *Thiomargarita* | >0.01 | 99 | *Alcaligenes* | >0.01 | 156 | *Rhodoblastus* | >0.01 |
| 43 | *Rhodospirillum* | >0.01 | 100 | *Azospira* | >0.01 | 157 | *Rhodomicrobium* | >0.01 |
| 44 | *Ralstonia* | >0.01 | 101 | *Bryobacter* | >0.01 | 158 | *Rhodopseudomonas* | >0.01 |
| 45 | *Thalassospira* | >0.01 | 102 | Candidatus Captivus | >0.01 | 159 | *Sphingomonas* | >0.01 |
| 46 | *Massilia* | >0.01 | 103 | *Dickeya* | >0.01 | 160 | *Staphylothermus* | >0.01 |
| 47 | *Bacillus* | >0.01 | 104 | *Ferrimicrobium* | >0.01 | 161 | *Synechococcus* | >0.01 |
| 48 | Candidatus Nitrosocaldus | >0.01 | 105 | *Ferroplasma* | >0.01 | 162 | *Telmatobacter* | >0.01 |
| 49 | *Roseomonas* | >0.01 | 106 | *Granulicella* | >0.01 | 163 | *Tepidimonas* | >0.01 |
| 50 | *Halomonas* | >0.01 | 107 | *Herminiimonas* | >0.01 | 164 | *Terriglobus* | >0.01 |
| 51 | *Neisseria* | >0.01 | 108 | *Hydrogenophaga* | >0.01 | 165 | *Thermithiobacillus* | >0.01 |
| 52 | *Simplicispira* | >0.01 | 109 | *Ignicoccus* | >0.01 | 166 | *Thermoanaerobacterium* | >0.01 |
| 53 | MWH-UniP1 aquatic group | >0.01 | 110 | *Inhella* | >0.01 | 167 | *Thermodesulforhabdus* | >0.01 |
| 54 | Burkholderia | >0.01 | 111 | *Marinicella* | >0.01 | 168 | *Thiohalomonas* | >0.01 |
| 55 | Candidatus Micrarchaeum acidiphilum ARMAN-2 | >0.01 | 112 | *Methylotenera* | >0.01 | 169 | *Tuberibacillus* | >0.01 |
| 56 | *Sutterella* | >0.01 | 113 | *Microbacterium* | >0.01 | 170 | *Vibrio* | >0.01 |
| 57 | *Aeropyrum* | >0.01 | 114 | *Microvirgula* | >0.01 |  |  |  |

**Table S2. Hydrogen metabolic proteins in *Hydrogenobaculum* sp. identified by NCBI search.**

| Description | Found |
| --- | --- |
| HypB | V |
| HupH | V |
| HypF | V |
| HypA | V |
| HypE | V |
| HypD | V |
| HypC/HupF | V |
| Hydrogenase maturation protease | V |
| Ni/Fe-hydrogenase, b-type cytochrome subunit | V |
| Nickel-dependent hydrogenase large subunit | V |
| HydA | V |
| Fe-S-cluster-containing hydrogenase subunit | V |
| Hydrogenase 4 membrane component (E) |  |
| Cytochrome-c3 hydrogenase |  |
| Ni,Fe-hydrogenase I large subunit |  |
| Ni,Fe-hydrogenase III large subunit |  |
| Hydrogenase 2 maturation peptidase |  |

**Table S3. Physiological features of major species or strains in the nine dominant microbial genera recovered from the SHP hot spring.**

| Rank | Assigned domain | Assigned genus | Major subgroup | Former name | *RA_contig_* (%) | Growth temperature (ºC) | Oxygen |
| --- | --- | --- | --- | --- | --- | --- | --- |
| 1 | **Bacteria** | ***Hydrogenobaculum*** |  |  | **5.73** |  |  |
|  |  |  | str. Y04AAS1  str. SN |  | 2.90  2.83 | 54 ^(1), *^  Unknown ^#^ | 4 ^(1)^,^*^  Unknown ^#^ |
| 2 | **Archaea** | ***Vulcanisaeta*** |  |  | **3.64** |  |  |
|  |  |  | *V. distributa*  *V. moutnovskia* |  | 2.42  1.22 | 70 – 99 ^(2)^  60 – 98 ^(3)^ | - ^(2)^  - ^(3)^ |
| 3 | **Archaea** | ***Thermoproteus*** |  |  | **2.82** |  |  |
|  |  |  | *T. uzoniensis*  *T. tenax* |  | 2.45  0.37 | 74 – 102 ^(4)^  opt. 86 ^(5)^ | - ^(4)^  - ^(5)^ |
| 4 | **Archaea** | ***Caldisphaera*** |  |  | **2.66** |  |  |
|  |  |  | *C. lagunensis* |  | 2.66 | 45 – 80 ^(6)^ | - ^(6)^ |
| 5 | **Archaea** | ***Sulfolobus*** |  |  | **2.43** |  |  |
|  |  |  | *S. tokodaii*  *S. solfataricus*  *S. acidocaldarius* | *Sulfolobus* sp. str. 7 ^(6)^ | 1.65  0.43  0.20 | 75 – 80 ^(6)^  70 – 80 ^(7)^  opt. 75 – 80 ^(8)^ | + ^(6)^  + ^(7)^  + ^(8)^ |
| 6 | **Archaea** | ***Caldivirga*** |  |  | **2.39** |  |  |
|  |  |  | *C. maquilingensis* |  | 2.39 | 60 – 92 ^(10)^ | - ^(10)^ |
| 7 | **Bacteria** | ***Acidithiobacillus*** |  | ***Thiobacillus*** ^(10)^ | **2.17** |  |  |
|  |  |  | *A. caldus*  *A. ferrooxidans* | *T. caldus* ^(10)^  *T. ferrooxidans* ^(10)^ | 1.60  0.39 | 32 – 52 ^(11)^  10 – 37 ^(12)^ | -/+ ^(11)^  -/+ ^(12)^ |
| 8 | **Bacteria** | ***Thiomonas*** |  |  | **1.66** |  |  |
|  |  |  | *T. arsenitoxydans*  *T. intermedia* | *Thiomonas* str. 3As ^(13)^ | 0.88  0.74 | 30 ^(13)^  30 – 35 ^(14)^ | + ^(13)^  + ^(14)^ |
| 9 | **Archaea** | ***Metallosphaera*** |  |  | **1.50** |  |  |
|  |  |  | *M. sedula* |  | 1.44 | 50 – 80 ^(15)^ | + ^(15)^ |

Superscript numbers (1 to 15) are reference numbers. For details, refer to the original reference listed in the Reference section of this supplementary document.

^#^ Physiology of these strains or species apparently not reported.

^*^ Optimal temperature and pH under the growth conditions described by Aguiar *et al.* were 54 ºC and 4.0, respectively (16). Later microbial ecology studies designated *Hydrogenobaculum* sp. Y04AAS1 as the dominant microbe in Dragon Spring (water temperature: 70 ~ 72 ºC; pH: 3.1), One Hundred Spring (water temperature: 73 ºC; pH: 3.5) and Norris Geyser (water temperature: 65 ºC; pH: 3; 17). In addition, Y04AAS1 dominated in SHP (water temperature: 69 ºC; pH: 2.5). Together, the report suggested *Hydrogenobaculum* sp. Y04AAS1 could thrive in a higher temperature than previous study suggested.

**Table S4. Mapping DSS raw reads *Hydrogenobaculum* sp. Y04AAS1 or *E. coli* DH10B with various length fraction and similarity fraction settings.**

|  | Run 1 | Run 2 | Run 3 | Run 4 | Run 5 |
| --- | --- | --- | --- | --- | --- |
| Reference | *Hydrogenobaculum* sp. Y04AAS1 | *Hydrogenobaculum* sp. Y04AAS1 | *Hydrogenobaculum* sp. Y04AAS1 | *Escherichia coli* DH10B | *Escherichia coli* DH10B |
| Length fraction | 0.9 | 0.9 | 0.5 | 0.9 | 0.5 |
| Similarity fraction | 0.95 | 0.7 | 0.8 | 0.95 | 0.8 |
| Global alignment | Yes | Yes | Yes | Yes | Yes |
| Reference length (bp) | 1,559,514 | 1,559,514 | 1,559,514 | 4,686,137 | 4,686,137 |
| GC (%) | 34.85 | 34.85 | 34.85 | 50.78 | 50.78 |
| Consensus length (bp) | 186,983 | 1,512,361 | 1,495,757 | 582.597 | 1,062,031 |
| Reference covered (%) | 0.12 | 0.97 | 0.96 | 0.12 | 0.23 |
| Minimum coverage (bp) | 0 | 0 | 0 | 0 | 0 |
| Maximum coverage (bp) | 40,056 | 189,756 | 172,089 | 92 | 36,901 |
| Average coverage (bp) | 158.14 | 11,091.56 | 10,872.79 | 0.18 | 24.18 |

**Table S5. Comparison of KEGG database and previous studies (reference for Figure S3).**

| **Pathway**^a^ | **Assigned Genus** | **Subgroup** | **In KEGG^b^** | **In Ref.** | **Reference for reaction** |
| --- | --- | --- | --- | --- | --- |
| **Dissimilatory nitrate reduction**  NO_3_^-^🡪NO_2_^-^🡪NH_3_ | *Acidithiobacillus* | *A. caldus* | complete | complete | You et al., 2011 Journal of Genetics and Genomics |
|  | *Thiomonas* | *T.* *arsenitoxydans* | complete | **incomplete** | Arsène-Ploetze 2010 PLoS Genetics |
| **Assimilatory nitrate reduction**  NO_3_^-^🡪NO_2_^-^🡪NH_3_ | *Thiomonas* | *T.* *arsenitoxydans* | incomplete | incomplete | Arsène-Ploetze 2010 PLoS Genetics |
| **Denitrification**  NO_3_^-^🡪NO_2_^-^🡪NO🡪N_2_O🡪N_2_ | *Hydrogenobaculum* | str. Y04SAA1 | incomplete | **complete** | Romano et al., 2013 Applied and Environmental Microbiology |
|  |  |  |  |  | Reysenbach et al., 2009 Journal of Bacteria |
|  | *Thiomonas* | *T.* *arsenitoxydans* | incomplete | incomplete | Arsène-Ploetze 2010 PLoS Genetics |
| **Nitrogen fixation** | *Acidithiobacillus* | *A. ferrooxidans* | complete | complete | Levicán et al., 2008 BMC Genomics |
| **Calvin cycle** | *Acidithiobacillus* | *A. caldus* | incomplete | **complete (unusual)** | You et al., 2011 Journal of Genetics and Genomics |
|  |  | *A. ferrooxidans* | incomplete | **complete (unusual)** | Osorio et al., 2013 Applied and Environmental Microbiology |
|  |  |  |  |  | Levicán et al., 2008 BMC Genomics |
|  | *Thiomonas* | *T.* *arsenitoxydans* | incomplete | **complete** | Arsène-Ploetze 2010 PLoS Genetics |
| **Dicarboxylate-hydroxybutyrate cycle** | *Thermoproteus* | *T. uzoniensis* | incomplete | **complete** | Mardanov et al., 2011 Journal of Bacteriology |
|  |  | *T. tenax* | incomplete | **complete** | Siebers et al., 2011 PLoS ONE |
| **Reductive citrate cycle** | *Hydrogenobaculum* | str. Y04SAA1 | complete | complete | Boyd et al., 2009 Applied and Environmental Microbiology |
|  |  |  |  |  | Reysenbach et al., 2009 Journal of Bacteriology |
|  |  | str. SN | complete | complete | Boyd et al., 2009 Applied and Environmental Microbiology |
|  | *Thermoproteus* | *T. uzoniensis* | incomplete | **complete** | Mardanov et al., 2011 Journal of Bacteriology |
|  |  | *T. tenax* | incomplete | **complete** | Siebers et al., 2007 Journal of Bacteriology |
|  | *Acidithiobacillus* | *A. ferrooxidans* | no info. | **complete** | Levicán et al., 2008 BMC Genomics |
| **Hydroxypropionate-hydroxybutyrate cycle** | *Sulfolobus* | *S. tokodaii* | complete | complete | Alber et al., 2006 Journal of Bacteriology |
|  |  | *S. solfataricus* | complete | complete | Ulas et al., 2012 PLoS ONE |
|  | *Metallosphaera* | *M. sedula* | complete | complete | Alber et al., 2006 Journal of Bacteriology |
| **SO_4_^2-^**🡪**APS**🡪**PAPS**🡪**SO_3_^2-^**🡪**S^2-^** | *Acidithiobacillus* | *A. ferrooxidans* | complete | complete | Valdés et al., 2003 BMC Genomics |
| **SO_4_^2-^🡨**🡪**APS🡨**🡪**SO_3_^2-^🡨**🡪**H_2_S** | *Vulcanisaeta* | *V. moutnovskia* | complete | complete | Gumerov et al., 2011 Journal of Bacteriology |
|  |  |  |  |  | Klenk et al., 1998 Nature |
|  | *Thiomonas* | *T. tenax* | complete | complete | Siebers et al., 2011 PLoS ONE |
|  | *Sulfolobus* | *S. tokodaii* | incomplete | **complete (unusual)** | Kawarabayasi et al., 2001 DNA Research |
|  |  | *S. solfataricus* | incomplete | **complete** | She et al., 2001 Proceedings of the National Academy of Sciences |
|  | *Caldivirga* | *C. maquilingensis* | complete | complete | Gumerov et al., 2011 Journal of Bacteriology |
|  |  |  |  |  | Klenk et al., 1998 Nature |
|  | *Acidithiobacillus* | *A. caldus* | incomplete | incomplete | Chen et al., 2012 PLoS ONE |
|  |  | *A. ferrooxidans* | incomplete | incomplete | Osorio et al., 2013 Applied and Environmental Microbiology |
|  | *Thiomonas* | *T.* *arsenitoxydans* | no info. | **complete** | Arsène-Ploetze 2010 PLoS Genetics |
|  | *Metallosphaera* | *M. sedula* | incomplete | **complete** | Auernik et al., 2008 Applied and Environmental Microbiology |
| **SOX system** | *Sulfolobus* | *S. tokodaii* | no info. | **complete** | Auernik and Kelly, 2008 Applied and Environmental Microbiology |
|  |  | *S. solfataricus* | no info. | **complete** | Auernik and Kelly, 2008 Applied and Environmental Microbiology |
|  |  | *S. acidocaldarius* | no info. | **complete** | Auernik and Kelly, 2008 Applied and Environmental Microbiology |
|  | *Acidithiobacillus* | *A. caldus* | complete | complete | Chen et al., 2012 PLoS ONE |
|  | *Thiomonas* | *T.* *arsenitoxydans* | complete | complete | Arsène-Ploetze 2010 PLoS Genetics |
|  | *Metallosphaera* | *M. sedula* | no info. | **complete** | Auernik and Kelly, 2008 Applied and Environmental Microbiology |
| **SO_3_^2-^**🡪**SO_4_^2-^** | *S. tokodaii* | *S. tokodaii* | no info. | **complete** | Kawarabayasi et al., 2001 DNA Research |
|  | *A. ferrooxidans* | *A. ferrooxidans* | no info. | **complete** | Osorio et al., 2013 Applied and Environmental Microbiology |
| **H_2_S**🡪🡪**S_n_** | *Acidithiobacillus* | *A. caldus* | complete | complete | Chen et al., 2012 PLoS ONE |
|  |  | *A. ferrooxidans* | complete | complete | Acosta et al., 2005. OMICS: A Journal of Integrative Biology |
|  | *Thiomonas* | *T.* *arsenitoxydans* | complete | complete | Arsène-Ploetze 2010 PLoS Genetics |
|  | *Metallosphaera* | *M. sedula* | complete | complete | Auernik and Kelly, 2008 Applied and Environmental Microbiology |
| **S_n_**🡪🡪**H_2_S** | *Thermoproteus* | *T. tenax* | no info. | **complete** | Siebers et al., 2011 PLoS ONE |
|  | *Acidithiobacillus* | *A. caldus* | no info. | **complete** | Chen et al., 2012 PLoS ONE |
| **S_0_**🡪**H_2_S**  sulfur oxygenase/reductase | *Thermoproteus* | *T. uzoniensis* | no info. | **complete** | Mardanov et al., 2011 Journal of Bacteriology |
|  |  | *T. tenax* | no info. | **complete** | Siebers et al., 2011 PLoS ONE |
|  | *Acidithiobacillus* | *A. caldus* | no info. | **complete** | Chen et al., 2012 PLoS ONE |
| **H_2_S**🡪**S_0_** | *Sulfolobus* | *S. tokodaii* | complete | complete | Kawarabayasi et al., 2001 DNA Research |
|  | *Acidithiobacillus* | *A. caldus* | no info. | **complete** | Chen et al., 2012 PLoS ONE |
|  |  | *A. ferrooxidans* | no info. | **complete** | Valdés et al., 2003 BMC Genomics |
|  | *Metallosphaera* | *M. sedula* | no info. | **complete** | Huber et al., 1989 Systematic and Applied Microbiology |
| **S_0_**🡪**SO_3_^2-^** | *Sulfolobus* | *S. solfataricus* | no info. | **complete** | She et al., 2001 Proceedings of the National Academy of Sciences |
|  | *Acidithiobacillus* | *A. ferrooxidans* | no info. | **complete** | Osorio et al., 2013 Applied and Environmental Microbiology |
| **S_4_O_6_^2-^**🡪**S_2_O_3_^2-^ +SO_4_^2-^+S_0_**  tetrathionate hydrolases  (TetH, 4THases) | *Sulfolobus* | *S. tokodaii* | no info. | **complete** | Auernik and Kelly, 2008 Applied and Environmental Microbiology |
|  | *Acidithiobacillus* | *A. caldus* | no info. | **complete**  **(unusual)** | Chen et al., 2012 PLoS ONE |
|  |  |  |  |  | Bugaytsova and Lindstrom et al., 2004 European Journal of Biochemistry |
|  |  | *A. ferrooxidans* | no info. | **complete** | Kanao et al., 2007 Journal of Biotechnology |
|  |  |  |  |  | Additional referance listed in Bugaytsova and Lindstrom et al., 2004 European Journal of Biochemistry |
|  | *Metallosphaera* | *M. sedula* | no info. | **complete** | Auernik and Kelly, 2008 Applied and Environmental Microbiology |
| **S_2_O_3_^2-^**🡪**S_4_O_6_^2-^**  TQO (DoxDA) | *Sulfolobus* | *S. tokodaii* | complete | complete | Kawarabayasi et al., 2001 DNA Research |
|  |  |  |  |  | Auernik and Kelly, 2008 Applied and Environmental Microbiology |
|  |  | *S. solfataricus* | complete | complete | Auernik and Kelly, 2008 Applied and Environmental Microbiology |
|  | *Acidithiobacillus* | *A. caldus* | complete | complete | Chen et al., 2012 PLoS ONE |
|  | *Metallosphaera* | *M. sedula* | complete | complete | Auernik and Kelly, 2008 Applied and Environmental Microbiology |
| **S_2_O_3_^2-^**🡪**SO_4_^-^** | *Acidithiobacillus* | *A. caldus* | complete | complete | Chen et al., 2012 PLoS ONE |

^a^Pathway names were those recorded in KEGG database (Release 72.0, Oct 1^st^ 2014). If no formal name was available, reaction equation was used as a name.

^b^Release 72.0, Oct 1^st^ 2014. “No info.”: no enzyme listed in reference pathway for the specific microbe.

**Table S6. CRISPR-like arrays detected in the SHP hot spring metagenome.**

| Species or strain | No. arrays assigned |
| --- | --- |
| *Hydrogenobaculum* sp. OH | 1 |
| *Sulfolobus islandicus* | 6 |
| *Metallosphaera sedula* | 6 |
| *Shewanella sp.* W3-18-1 | 1 |
| *Pyrobaculum calidifontis* | 1 |
| *Caldisericum exile* | 107 |

**Table S7. Viral sequences identified in the SHP hot spring metagenome.**

| No. | Phage |
| --- | --- |
| 1 | *Pseudomonas* phage AF |
| 2 | *Acidianus* spindle-shaped virus 1 |
| 3 | *Acidianus* two-tailed virus |
| 4 | *Sulfolobus* spindle-shaped virus 4 |
| 5 | *Sulfolobus* spindle-shaped virus 7 |

**Table S8. Statistics regarding DNA direct sequencing (Illumina GS-FLX sequencer) and summary of assembly (Metavelvet).**

| Total reads | 557,415,266 |
| --- | --- |
| Total reads after quality trim^*^ | 548,895,370 |
| Read length (bp) | 101 |
| Read length after quality trim^*^ | 98 |
| Number of contigs | 126,849 |
| Average contig length (bp) | 478.4 |
| Max / min contig length (bp) | 313819 / 149 |
| Contig ≧ 300 bp, number (percentage) | 43,015 (23.8%) |
| Contig ≧ 1 kb, number (percentage) | 10,014 (5.5%) |
| Contig ≧ 40 kb, number (percentage) | 112 (0.06%) |
| N50 | 1191 |
| Number of “N” | 1,811,850 |
| N-ratio | 0.02 |

^*^Quality trim criteria: min length = 35 bp, error probability < 0.05

**Table S9. Sequencing summary of 1485 fosmid clones and assembly summary (MetaVelevet).**

| Total reads | 224,299,204 |
| --- | --- |
| Total reads after quality trim^*^ | 219,083,057 |
| Reads after vector removal | 159,891,223 |
| Percentage of vector in total reads after quality trim^*^ | 27% |
| Read length (bp) | 101 |
| Read length after quality trim (bp)^*^ | 94.4 |
| Read length after vector removal (bp) | 93.6 |
| Number of contigs | 52,221 |
| Average contig length (bp) | 559.45 |
| Max / min contig length (bp) | 325,311 / 129 |
| Contig ≧ 300 bp, number (percentage) | 12,648 (24.2%) |
| Contig ≧ 1 kb, number (percentage) | 3,137 (6.0%) |
| Contig ≧ 40 kb, number (percentage) | 45 (0.09%) |
| N50 | 2,693 |
| Number of “N” | 48,975 |
| N-ratio | 0.002 |

^*^Quality trim criteria: min length = 35 bp, error probability < 0.05

**Figure S1. Relative abundances of metagenomic information for each microbial genus in SHP.** Dotted line represents the threshold for selecting information-rich microbial genera.

**Figure S2. Line plot of functional categories of COGs for the three metagenomes.** NNPLN, National Natural Park Los Nevados (Colombia); YNP, Yellowstone National Park (USA); SHP, Shi-Huang-Ping (Taiwan).

**Figure S3. Energy metabolism networks of dominant microbes.** Metabolic network was generated based on KEGG reference pathways. Numbers indicate microbial genera capable of conducting biochemical reactions; light green notes indicate metabolic abilities within the group. 1: *Hydrogenobaculum*, 2: *Vulcanisaeta*, 3: *Thermoproteus*, 4: *Caldisphaera*, 5: *Sulfolobus*, 6: *Caldivirga*, 7: *Acidithiobacillus*, 8: *Thiomonas*, 9: *Metallosphaera*. Red group numbers are enzymes responsible for that specific reaction detected in our metagenomic data. Blue group numbers or lines mark reactions not included in KEGG reference pathways, but discussed or studied in previous reports. Light blue line indicates reaction byproducts for the metabolic pathways. Blue triangle indicates reactions studied in previous reports (references for those reactions are in Table S6).

**Figure S4. Percent identity, coverage and e-value reported after blast CRISPR-like array.** The red line on the plot and the scale on left y-axis referred to coverage of the query; the blue line on the plot and the scale on left y-axis referred to the identity of the query length in alignment. The green line (refer to right y-axis for scale) was the actual e-value reported after blast.

**REFERENCES**

1. Ferrera I, Longhorn S, Banta AB, Liu Y, Preston D, Reysenbach A-L. **Diversity of 16S rRNA gene, ITS region and *aclB* gene of the Aquificales.** *Extremophiles.* 2007;**11**:57-64.

2. Itoh T, Sukuki K-i, Nakase T. ***Vulcanisaeta distributa* gen. nov., sp. nov., and *Vulcanisaeta souniana* sp. nov., novel hyperthermophilic, rod-shaped crenarchaeotes isolated from hot springs in Japan.** *Int J Syst Evol Microbiol.* 2002;**52**:1097-1104.

2. Gumerov VM, Mardanov AV, Beletsky AV, Prokofeva MI, Bonch-Osmolovskaya EA, Ravin NV, Skryabin K. **Complete genome sequence of “*Vulcanisaeta moutnovskia*” Strain 768-28, a novel member of the hyperthermophilic crenarchaeal genus *Vulcanisaeta*.** *J Bacteriol.* 2011;**193**:2355-2356.

3. Bonch-Osmolovskaya EA, Miroshnichenko ML, Kostrikina NA, Chernych NA, Zavarzin GA. ***Thermoproteus uzoniensis* sp. nov., a new extremely thermophilic archaebacterium from Kamchatka continental hot springs.** *Arch Microbiol*. 1990;**154**:556-559

4. Zillig W, Stetter KO, Schäfer W, Janekovic D, Wunderl S, Holz I, Palm P. ***Thermoproteales*: A novel type of extremely thermoacidophilic anaerobic archaebacteria isolated from Icelandic solfataras.** *Zentralblatt für Bakteriologie Mikrobiologie und Hygiene: I Abt Originale C.* 1981;**2**:205-227.

5. Itoh T, Sanchez PC, Nakase T. ***Caldisphaera lagunensis* gen. nov., sp. nov., a novel thermoacidophilic crenarchaeote isolated from a hot spring at Mt Maquiling, Philippines.** Int J Syst Evol Microbiol. 2003;**53**:1149-1154.

6. Suzuki T, Iwasaki T, Uzawa T, Hara K, Nemoto N, Kon T, Ueki T, Yamagishi A, Oshima T. ***Sulfolobus tokodaii* sp. nov. (f. *Sulfolobus* sp. strain 7), a new member of the genus *Sulfolobus* isolated from Beppu Hot Springs, Japan.** *Extremophiles.* 2002;**6**:39-44.

7. Zillig W, Stetter KO, Wunderl S, Schulz W, Priess H, Scholz I. **The *Sulfolobus-“Caldariella”* group: Taxonomy on the basis of the structure of DNA-dependent RNA polymerases.** *Arch Microbiol.* 1980;125:259-269.

8. Broc TD, Brock KM, Belly RT, Weiss RL. ***Sulfolobus*: a new genus of sulfur oxidising bacteria living at low pH and high temperature.** *Arch Microbiol*. 1972;**84**:54-68.

9. Itoh T, Suzuki K-i, Sanchez PC, Nakase T. ***Caldivirga maquilingensis* gen. nov., sp. nov., a new genus of rod-shaped crenarchaeote isolated from a hot spring in the Philippines.** *Int J Syst Bacteriol.* 1999;**49**:1157-1163.

10. Kelly DP, Wood AP. **Reclassification of some species of *Thiobacillus* to the newly designated genera *Acidithiobacillus* gen. nov., *Halothiobacillus* gen. nov. and *Thermithiobacillus* gen.** *Int J Syst Evol Microbiol.* 2000;**50**:511-516.

11. Hallberg KB, Lindström EB. **Characterization of *Thiobacillus* *caldus* sp. nov., a moderately thermophilic acidophile.** *Micriobiology.* 1994;**140**:3451-3456.

12. Waksman SA, Joffe JS. **Microorganisms concerned in the oxidation of sulfur in the soil. II. *Thiobacillus thiooxidans*, a new sulfur-oxidizing organism isolated from the soil.** *J Bacteriol.* 1922;**7**:239-256.

13. Slyemi D, Moinier D, Brochier-Armanet C, Bonnefoy V, Johnson DB. **Characteristics of a phylogenetically ambiguous, arsenic-oxidizing *Thiomonas* sp., *Thiomonas* *arsenitoxydans* strain 3As^T^ sp. nov.** *Arch Microbiol.* 2011;**193**:439-449.

14. London J. ***Thiobacillus intermedius* nov. sp. A novel type of facultative autotroph.** *Archiv fur Mikrobiologie.* 1963;**46**:329–337.

15. Huber G, Spinnler C, Gambacorta A, Stetter KO. ***Metallosphaera* *sedula* gen. and sp. nov. represents a new genus of aerobic, metal-mobilizing, thermoacidophilic archaebacteria.** *Syst Appl Microbiol.* 1989;**12**:38-47.

16. Aguiar P, Beveridge TJ, Reysenbach A-L. ***Sulfurihydrogenibium* *azorense*, sp. nov., a thermophilic hydrogen-oxidizing microaerophile from terrestrial hot springs in the Azores.** *Int J Syst Evol Microbiol.* 2004;**54**:33–39

17. Inskeep WP, Jay ZJ, Tringe SG, Herrgård MJ, Rusch DB and YNP Metagenome Project Steering Committee and Working Group Members: **The YNP Metagenome Project: Environmental Parameters Responsible for Microbial Distribution in the Yellowstone Geothermal Ecosystem.** Front Microbiol 2013;67:1-6

18. SF Stoddard, BJ Smith, R Hein, BRK Roller and TM Schmidt: ***rrn*DB: improved tools for interpreting rRNA gene abundance in bacteria and archaea and a new foundation for future development.** Nucleic Acids Res. 2015;43:D593-8
